# Supplementary figures and images for: Plasma SerpinA5 in conjunction with uterine artery pulsatility index and clinical risk factor for the early prediction of preeclampsia
Source: PLoS One. 2021 Oct 14;16(10):e0258541. doi: 10.1371/journal.pone.0258541 (PMC8516267; doi:10.1371/journal.pone.0258541)

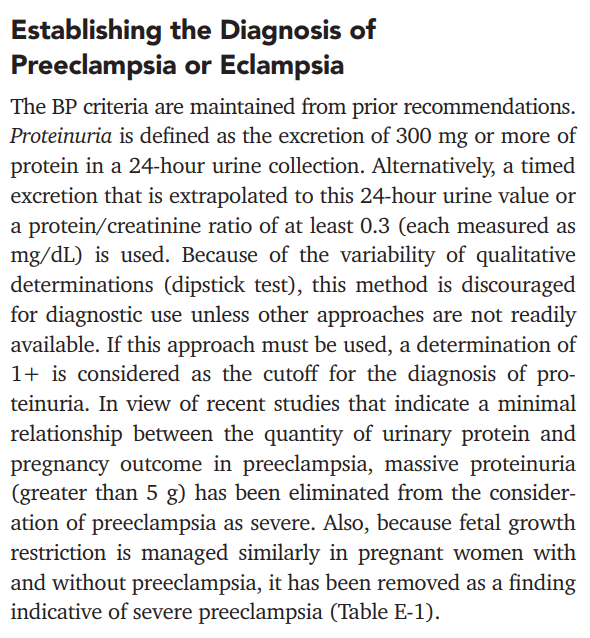


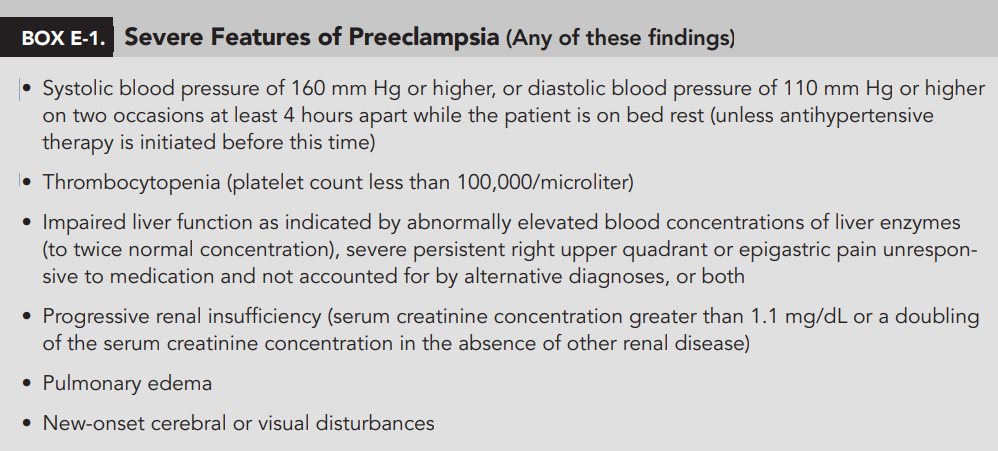

Supplement: S1 File — (DOCX) [file pone.0258541.s001.docx]
