# Supplementary material for: Plasma SerpinA5 in conjunction with uterine artery pulsatility index and clinical risk factor for the early prediction of preeclampsia
Source: PLoS One. 2021 Oct 14;16(10):e0258541. doi: 10.1371/journal.pone.0258541 (PMC8516267; doi:10.1371/journal.pone.0258541)
Supplement: Attachment — Submitted filename: Response to Reviewers.docx [file pone.0258541.s005.docx]

Aug 20 2021

Dear Ph.D. Maurizio Mandalà and other Editors：

Thank you very much for your decision letter and advice on our manuscript (PONE-D-21-13434), entitled “*Plasma serpinA5 in conjunction with uterine artery pulsatility index and clinical risk factor for the early prediction of preeclampsia*”. We also thank the reviewers a lot for their insightful and positive comments and suggestions. Accordingly, we have revised the manuscript, and all amendments are highlighted in red. Point-by-point responses to the comments are listed below this letter. We hope that the revised manuscript is acceptable for publication in your journal, and we look forward to hearing from you soon.

Best wishes.

Yours sincerely,

Yonggang Zhang

Note:“All relevant data are within the paper and its Supporting Information files.”

**Replies to editors:**
When submitting your revision, we need you to address these additional requirements.
1. Please ensure that your manuscript meets PLOS ONE's style requirements, including those for file naming. The PLOS ONE style templates can be found at
<https://journals.plos.org/plosone/s/file?id=wjVg/PLOSOne_formatting_sample_main_body.pdf> and
<https://journals.plos.org/plosone/s/file?id=ba62/PLOSOne_formatting_sample_title_authors_affiliations.pdf>

**Response**: Thank you very much for proposing these requirements. We have carefully readed these downloaded files and modified the manuscript as the files required. We hope these modifications can be satisfactory to you. If there are still deficiencies, we will accept suggestion modestly!

2. In your Data Availability statement, you have not specified where the minimal data set underlying the results described in your manuscript can be found. PLOS defines a study's minimal data set as the underlying data used to reach the conclusions drawn in the manuscript and any additional data required to replicate the reported study findings in their entirety. All PLOS journals require that the minimal data set be made fully available. For more information about our data policy, please see <http://journals.plos.org/plosone/s/data-availability.>
"Upon re-submitting your revised manuscript, please upload your study’s minimal underlying data set as either Supporting Information files or to a stable, public repository and include the relevant URLs, DOIs, or accession numbers within your revised cover letter. For a list of acceptable repositories, please see <http://journals.plos.org/plosone/s/data-availability#loc-recommended-repositories.> Any potentially identifying patient information must be fully anonymized.
Important: If there are ethical or legal restrictions to sharing your data publicly, please explain these restrictions in detail. Please see our guidelines for more information on what we consider unacceptable restrictions to publicly sharing data: <http://journals.plos.org/plosone/s/data-availability#loc-unacceptable-data-access-restrictions.> Note that it is not acceptable for the authors to be the sole named individuals responsible for ensuring data access.
We will update your Data Availability statement to reflect the information you provide in your cover letter.

**Response**: Thank you very much for proposing these requirements. We have provided minimal data set in Supporting Information files as required and modified the statement: All relevant data are within the paper and its Supporting Information files.

3. PLOS requires an ORCID iD for the corresponding author in Editorial Manager on papers submitted after December 6th, 2016. Please ensure that you have an ORCID iD and that it is validated in Editorial Manager. To do this, go to ‘Update my Information’ (in the upper left-hand corner of the main menu), and click on the Fetch/Validate link next to the ORCID field. This will take you to the ORCID site and allow you to create a new iD or authenticate a pre-existing iD in Editorial Manager. Please see the following video for instructions on linking an ORCID iD to your Editorial Manager account: <https://www.youtube.com/watch?v=_xcclfuvtxQ>

**Response:** Thank you very much for proposing these requirements. We have added the ORCID iD for the corresponding author in Editorial Manager.

4. Your ethics statement should only appear in the Methods section of your manuscript. If your ethics statement is written in any section besides the Methods, please delete it from any other section.

**Response:** We were apologized for not doing well enough. The ethics statement has only appeared in the Methods section. The ethics statement at the bottom of this paper has been deleted.

5. Please include captions for your Supporting Information files at the end of your manuscript, and update any in-text citations to match accordingly. Please see our Supporting Information guidelines for more information: <http://journals.plos.org/plosone/s/supporting-information.>

**Response:** Thank you very much for proposing the requirement. Supporting Information has been modified as required.

6.Please review your reference list to ensure that it is complete and correct. If you have cited papers that have been retracted, please include the rationale for doing so in the manuscript text, or remove these references and replace them with relevant current references. Any changes to the reference list should be mentioned in the rebuttal letter that accompanies your revised manuscript. If you need to cite a retracted article, indicate the article’s retracted status in the References list and also include a citation and full reference for the retraction notice.
**Response:** Thanks for your reminding, we have checked the all the references. And the cited references meet the above requirements


**Replies to Reviewers:**
Reviewer's Responses to Questions

1. Is the manuscript technically sound, and do the data support the conclusions?
The manuscript must describe a technically sound piece of scientific research with data that supports the conclusions. Experiments must have been conducted rigorously, with appropriate controls, replication, and sample sizes. The conclusions must be drawn appropriately based on the data presented.

Reviewer #1: Yes

Reviewer #2: Partly

**Response:** We would like to express our sincere gratitude to the reviewers for your constructive and positive comments.

 2. Has the statistical analysis been performed appropriately and rigorously?

Reviewer #1: Yes

Reviewer #2: Yes

**Response:** We would like to express our sincere gratitude to the reviewers for your constructive and positive comments.

3. Have the authors made all data underlying the findings in their manuscript fully available?
The [PLOS Data policy](http://www.plosone.org/static/policies.action#sharing) requires authors to make all data underlying the findings described in their manuscript fully available without restriction, with rare exception (please refer to the Data Availability Statement in the manuscript PDF file). The data should be provided as part of the manuscript or its supporting information, or deposited to a public repository. For example, in addition to summary statistics, the data points behind means, medians and variance measures should be available. If there are restrictions on publicly sharing data—e.g. participant privacy or use of data from a third party—those must be specified.

Reviewer #1: No

Reviewer #2: Yes

**Response:** We would like to express our sincere gratitude to the reviewers for your constructive and positive comments. We have added the corresponding other data in the supporting information.

4. Is the manuscript presented in an intelligible fashion and written in standard English?
PLOS ONE does not copyedit accepted manuscripts, so the language in submitted articles must be clear, correct, and unambiguous. Any typographical or grammatical errors should be corrected at revision, so please note any specific errors here.

Reviewer #1: Yes

Reviewer #2: Yes

**Response:** We would like to express our sincere gratitude to the reviewers for your constructive and positive comments.

**Replies to Reviewer 1**

Reviewer #1: The manuscript entitled “Plasma serpinA5 in conjunction with uterine artery pulsatility index and clinical risk factor for the early prediction of preeclampsia” evaluated the combined use of plasma protein SerpinA5, uterine artery doppler and clinical risk factors in the prediction of preeclampsia. The study is potentially interesting and well designed, but I have some suggestions before this paper is suitable to for publication as follows bellow.

**Response:**

Thank you very much for your positive comment on the study and insightful suggestion. We have revised the manuscript, point-by-point responses to the comments are listed below. I hope these replies would satisfy you.

1). Abstract: The objective needs to be clearer indicating which plasma protein was evaluated (SerpinA5). Material and methods and results must inform about the in vitro assay performed with TEV-1 cells.

**Response**:

Thank you for your helpful suggestion. We have made the relevant corrections as required (Page 2 Line 31 and Page 2 Lines 43-44 and Page 2 Lines 47-48).

2). Keywords: Remove the abbreviation “PE”.

Response: Thank you very much for your serious and meticulous work！

We apologize for our negligence. This correction has been made (Page 3 Line 56).

3). Material and methods:
Line 97: Inform the meaning of “ACOG”. Inform the other characteristics for classification within the preeclampsia group.
Line 115: Inform the proportion of cesarean and natural delivery in each group. Because the authors did not only standardize samples of cesarean deliveries, since natural delivery can alter the expression of some placental proteins.
Line 116: Remove a “)”. It's duplicated.
Lines 145-146: How many fields were evaluated per placenta and which augmentation used.
Line 179: Remove “and”.
**Response:** Thank you for your valuable suggestion.

The modifications have been made into the revised manuscript.

ACOG is American College of Obstetricians and Gynecologists (Page 4 Lines 87).

The other characteristics for classification within the preeclampsia group was showed in Supporting information S1 File from Ref 1 as below [1].

The proportion of cesarean and natural delivery in each group was showed in Table 1.

Per section and three sections each specimen and ten randomly selected fields were evaluated and no augmentation was used in immunohistochemistry (Page 6 Lines 137-138). and other repeated or incorrect wording was deleted (Page 5 Line 108 and Page 8 Line 172).

We hope that the revisions satisfy the above-mentioned requirements.

[1]. Hypertension in pregnancy. Report of the American College of Obstetricians and Gynecologists’ Task Force on Hypertension in Pregnancy. Obstet Gynecol. 2013 Nov;122(5):1122-1131.

4). Results:
Line 191: Rewrite the sentence. “UtA-PI of preeclampsia was signifcantly diferences than control”.
Subdivide the results into Clinical Characteristics, Proteomics and ELISA, SerpinA5 Immunohistochemistry, In vitro migration analysis, Prediction analysis and further describe each result. The description is superficial and does not adequately describe all results.
Figure captions are uninformative. Need to inform the statistical analysis, N sample, title of the figure. Ex.: What does MSP mean in figure 6? The authors need to graphically demonstrate the immunohistochemical analysis and in vitro migration test.
There are too many figures. Some results can be joined together to reduce the number of figures. (Ex: Figures 7 and 8).
**Response**: Thank you for your insightful and good suggestion.

The modifications have been made into the revised manuscript.

The average UtA-PI of preeclampsia was significantly higher than that control during the first trimester (Rewrite the sentence in Page 9 Lines 186-187).

The results have been subdivided into Clinical Characteristics, Proteomics and ELISA, SerpinA5 Immunohistochemistry, In vitro migration analysis, Prediction analysis (Page 8 Line 183 and Page 9 Lines 188, 195, 199, 206) and further described (Page 9 Lines 196,197, 200-205 and Page 10 Lines 207-209).

Figure captions and Figure have been revised as required.

MSP is level of macrophage stimulating protein (Page 19 Line 420).

Statistical analysis and N sample have been added into Figure legends 2-3(Page 19 Line 406-415). Titles of the figure have been added into Figure legends 1-6 (Page 19 Line 406-425).

The immunohistochemical analysis and in vitro migration test have been graphically presented in Fig 3, 4.

We hope that all the revisions satisfy the above-mentioned requirements.

5). Discussion
Lines 225-227: "Some substances (such as VEGF, FLT, and 226 ENG) from the placenta enter into the maternal circulation, resulting in endothelial cell injury and a series of clinical manifestations of PE." Misinterpretation. Does VEGF Cause Endothelial Cell Injury? sFlt1 and not FLT.
Authors need to better discuss their own results, including clinical data.

**Response:**

Thank you very much for your good suggestion. The modifications have been made to the revised manuscript (Page 11 Line 234). We apologize for our inaccurate language expression, actually, we mean it is sFlt1 and not FLT.
The discussion section has been modified (Page 12 Lines 268-271). We hope that the revisions satisfy your requirements.

**Replies to Reviewer 2**

Reviewer #2: In the present study, Yonggang Zhang and colleagues ……. They concluded that SerpinA5 may be a novel biomarker for preeclampsia in conjunction with uterine artery pulsatility index and clinical risk factor may be helpful for the early prediction of preeclampsia.
This is an interesting study addressing, however, an extensively investigated issue as biomarkers in early diagnosis of Preeclampsia. Nevertheless, SerpinA5 role and its correlation with UtA-PI and maternal factors in PE placentae could be of interest to Plos One readers.
There are several issues that the Authors must address before publication.
**Response:** Thank you very much for your positive comment on the study and insightful suggestion for further investigation. We have revised the manuscript, point-by-point responses to the comments are listed below. I hope these replies would satisfy you.

1) The Authors enrolled PE and controls pregnant women however no complete clinical information were reported (e.g. mode of delivery, therapy, fetal sex, placental weight…). Moreover, they must clarify clinical criteria used for PE diagnosis: gestational age at delivery are not significantly different however the fetal weight is significantly decreased in PE. Therefore, did you consider PE with and/or without fetal-placental compromission? Please clarify. Finally, the Authors did not mention the possibility of a fetal-placenta disease in the PE group as the presence of Fetal Growth Restriction. These informations are mandatory to properly analyze and interpret study results.

Response: Thank you for your insightful and valuable suggestion. I can't agree with you more. The amount and proportion of mode of delivery has been added Table 1. There was no difference in fetal sex between the two groups. Because it's not our focus, we're very apologetic that we didn't collect the data of the therapy, and placental weight.

Gestational age of collecting specimen is very important for the expression of plasma and placental factors. These factors are not only affected by pathological changes, but also may change greatly with gestational weeks. Therefore, the gestational weeks matching between control group and PE group is very important. As reported in a literature [2], unexplained preterm birth (PTL) is a better control group for PE group with small gestational weeks. So, in the control, including normal pregnancy group and PTL. Thus , the gestational age at delivery is not significantly different. And no Fetal Growth Restriction in PE group (Table1).

[2]. Zhou Y, Bianco K, Huang L, Nien JK, McMaster M, Romero R, Fisher SJ. Comparative analysis of maternal-fetal interface in preeclampsia and preterm labor. Cell Tissue Res. 2007 Sep;329(3):559-69.

2) Please add the number of patients included in table 1 and figures 2, 3, 4, 5, 6

Response: the number of patients has been added included in new Table 1 and Figure legends 2, 3(Page 19 Lines 406-415); and the assays in Figures 3, 4, 5 was repeated three times and described in Page 6 Lines 137-138 and Page 7 Line 159.

3) The Authors performed immunohistochemistry and invasive trial and they demonstrated that SerpinA5 is over-expressed in PE placentae and inhibit MSP leading to a suboptimal trophoblast invasion explaining part of PE pathogenesis. Do you refer to PE with abnormal placental development? Do you think that this mechanism started during the first weeks (first trimester, when you discovered higher plasma SerpinA5?) Why did you chose TEV-1 cells? IHC is a semiquantitative techniques and you cannot conclude that SerpinA5 is over-expressed. You should perform a q quantitative techniques such as Real Time PCR and ELISA assay. Please, clarify

**Response:**

Thank you for raising this issue.

Placental theory holds that preeclampsia is mainly caused by poor placental formation. Preeclampsia occurs after 20 weeks. However, there are differences in some plasma factors of placenta before 20 weeks of pregnancy or even during the first pregnancy. SerpinA5 is over-expressed in PE placentae after delivery (>20 weeks). The onset and development of preeclampsia is very complex, here we are just making a very simple preliminary exploration of SerpinA5 in PE. The main purpose of this study is to do prediction analysis for PE, not mechanism research for PE.

Hui Chen Feng et al [3]. transfected the primary trophoblast isolated from the placenta of early pregnancy with the viral vector of HPV E16 / E17 gene to immortalize the cells. The phenotypic identification methods such as immunohistochemistry, RT-PCR and western blotting showed that TEV-1 (human extravillous trophoblast cell line) cell line maintained all the markers of extravillous trophoblasts. Therefore, it can be said that the immortalized cell line. TEV-1 cells maintain the characteristics of extravillous trophoblasts and can express invasive proteins. TEV-1 cell line can be used as a cell model to study the physiological and functional characteristics of human extravillous trophoblasts [3].

Thank you for your positive comment on the study and insightful suggestion for further investigation. In the next mechanism research, we are planning a further study to demonstrate the relationship between placental expression of SerpinA5 and preeclampsia by western blot analysis, and we prepare to do more in vivo and in vitro mechanism research. In IHC analysis in the present study, 10 microscopic fields were randomly selected in each section. Image Pro-Plus 6.0 software, which is useful for localization and semi-quantitative analysis, was used to quantify the integrated optical densities (IODs) of the SerpinA5-positive reactions.

[3].Feng HC, Choy MY, Deng W, Wong HL, Lau WM, Cheung AN, Ngan HY, Tsao SW. Establishment and characterization of a human first-trimester extravillous trophoblast cell line (TEV-1). J Soc Gynecol Investig. 2005 May;12(4):e21-32.

4) Please, better specify why SerpinA5 is better than sFlt-1/PlGF (e.g. higher sensitivity and specificity? Earlier diagnosis?)

**Response:**

Thank you for your constructive suggestion

The results are need to be validated in larger numbers. but the prediction effectiveness of sigle plasma protein SerpinA5 is insufficient, and plasma protein alone(sFlt-1/PlGF) is not enough (N Engl J Med. 2016; 374(18):1785-1786.) As stated in ACOG 2013, the combination of multiple prediction means is required for PE prediction. This study combined with a new plasma protein SerpinA5, ultrasonic Doppler and clinical risk factors to predict preeclampsia, which provides a new way for the prediction of preeclampsia.

PLOS authors have the option to publish the peer review history of their article ([what does this mean?](https://journals.plos.org/plosone/s/editorial-and-peer-review-process#loc-peer-review-history)). If published, this will include your full peer review and any attached files.
If you choose “no”, your identity will remain anonymous but your review may still be made public.
Author Response: “Yes”, but we respect the choices of editors and reviewers.
